# Avidity‐Based Selection of Tissue‐Specific CAR‐T Cells from a Combinatorial Cellular Library of CARs

**DOI:** 10.1002/advs.202003091

**Published:** 2021-01-29

**Authors:** Peixiang Ma, Ping Ren, Chuyue Zhang, Jiaxing Tang, Zheng Yu, Xuekai Zhu, Kun Fan, Guanglei Li, Wei Zhu, Wei Sang, Chenyu Min, Wenzhang Chen, Xingxu Huang, Guang Yang, Richard A. Lerner

**Affiliations:** ^1^ Shanghai Institute for Advanced Immunochemical Studies ShanghaiTech University Shanghai 201210 China; ^2^ School of Life Science and Technology ShanghaiTech University Shanghai 201210 China; ^3^ Institute of Biochemistry and Cell Biology Shanghai Institutes for Biological Sciences Chinese Academy of Sciences Shanghai 200031 China; ^4^ University of Chinese Academy of Sciences Beijing 100049 China; ^5^ Department of Hematology The Affiliated Hospital of Xuzhou Medical University Institute of Hematology Xuzhou Medical University Xuzhou 221000 China; ^6^ Velox Pharmaceuticals Changzhou 213000 China; ^7^ Department of Chemistry Scripps Research Institute La Jolla CA 92037 USA

**Keywords:** CD38, chimeric antigen receptor, combinatorial antibody library, tumor‐associated antigen

## Abstract

Using T‐cell chimeric antigen receptors (CAR‐T) to activate and redirect T cells to tumors expressing the cognate antigen represents a powerful approach in cancer therapy. However, normal tissues with low expression of tumor‐associated antigens (TAAs) can be mistargeted, resulting in severe side effects. An approach using a collection of T cells expressing a diverse, 10^6^‐member combinatorial cellular library of CARs, in which members can be specifically enriched based on avidity for cell membrane antigens, is reported. Using CD38 as the target antigen, an efficient and effective selection of CARs specifically recognizing CD38^+^ tumor cells is demonstrated. These selected CAR‐T's produce cytokines known to be associated with T cell activation in a CD38 expression‐dependent manner. This avidity‐based selection endows the engineered T cells with minimal off‐tumor effects, while retaining robust antitumor efficacy both in vitro and in vivo. The described method may facilitate the application of CAR‐T therapy to TAAs previously considered undruggable.

## Graphical abstract

A novel polyclonal combinatorial cellular library of chimeric antigen receptors provides a comprehensive coverage of immune responses to specific antigens in vitro. An efficient and effective avidity‐based selection strategy takes advantage of paracrine signaling systems in cell–cell interactions, which endow the engineered T cells with robust antitumor efficacy both in vitro and in vivo with minimal on‐target off‐tumor effects.

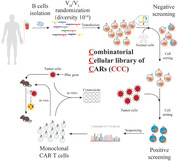

## 1 Introduction

The aim of cancer immunotherapy is to boost a patient's immune response to their tumor cells.^[^ ^1^ ^]^ In a promising approach, genetically engineered T cells can be activated and redirected to eradicate target tumor cells.^[^ ^2^ ^]^ This represents a powerful new modality in the fight against cancer. For example, in clinical trials for leukemia, treatment with T cells bearing chimeric antigen receptors targeting CD19 has been shown to result in impressive and sustained response rates.^[^ ^3^ ^]^ In addition, almost all multiple myeloma patients in a clinical trial with T‐cell chimeric antigen receptors (CAR‐T) cells targeting the B cell maturation antigen (BCMA) showed a complete or nearly complete response.^[^ ^4^ ^]^

However, one major hurdle to this approach is the fact that most antigens found on tumors are tumor‐associated (TAA) rather than tumor‐specific (TSA), i.e., they are expressed at some level on both tumor and normal cells.^[^ ^5^ ^]^ Despite the fact that there is often a dramatic difference in the level of expression, even minimal expression of the cognate antigen can lead to a T cell attack of normal tissue which triggers a massive cytokine release and on‐target off‐tumor toxicity.^[^ ^6^ ^]^ Life threatening toxicity was observed in clinical trials with CAR‐T cells targeting carbonic anhydrase IX (CAIX), ERBB2/HER2, or CEACAM5 because of recognition of their cognate antigens on normal bile duct and lung epithelia.^[^ ^7^ ^]^ Clearly, an efficient and safe therapeutic T cell therapy requires sophisticated tuning and precise aiming of T cells to engineer proper immune cell responses.

The specificity of CAR‐T is mediated by specific recognition between the antigen on the target cell surface and the engineered antibody on the CAR‐T cell surface. Thus, avidity engineering of CARs provides a plausible way to increase the antitumor potency of CAR‐T cells, and at the same time, modulates on‐target off‐tumor toxicity. Importantly, because T cell recognition and activation is a complex process, such engineering is best when it takes place in the context of antigen presentation on the tumor cell surface.^[^ ^8^ ^]^ Using the power of selection from large numbers, it is possible to adjust the avidity of CARs to obtain CAR‐T cells that discriminate between low antigen density on normal cells and higher antigen load on tumor cells.^[^ ^6^ ^]^ Previous efforts to adjust CAR avidity have been largely restricted to analyzing single chain combinatorial antibody (scFv) antibodies of different binding affinities with purified target antigen in a CAR‐T system.^[^ ^9^, ^10^, ^11^ ^]^ But we envisioned that the most straightforward and effective approach may be to create a diverse population of chimeric T cells large enough to contain the majority of all possible CARs. Desired members can then be efficiently enriched and selected in a coculture containing a second antigen‐expressing cell, using methods similar to affinity panning and maturation in the combinatorial antibody library approach.^[^ ^12^ ^]^

We chose to pursue this approach using CD38 as the target antigen and synNotch as the cell–cell interaction reporter system. CD38 is a validated drug target on multiple myeloma (MM) cells, as well as on a subset of hematologic tumors. In clinical studies, administration of Daratumumab, an anti‐CD38 monoclonal antibody, showed little or no toxicity at moderate doses, but led to increased risk of liver damage at high doses.^[^ ^13^ ^]^ An appealing and more specific immunotherapy strategy for MM was proposed via the adoptive transfer of CD38‐targeting cytotoxic T cells.^[^ ^14^ ^]^ One major concern in CD38‐targeted therapy is the wide expression of CD38 in a range of different tissues. In addition to tumor cells, CD38 is also highly expressed on normal plasma, lymphoid, and myeloid cells, as well as on red blood cells and platelets.^[^ ^15^ ^]^ In hematopoietic cells, natural killer (NK) cells express higher levels of CD38 than subpopulations of B and T cells.^[^ ^16^ ^]^ Cells expressing CD38 are also distributed in tissues of non‐hematopoietic origin including prostatic epithelial cells, pancreatic islet cells, and renal tubules cells, as well as in the perikaryal and dendrites of some neurons.^[^ ^17^ ^]^ As expected, the anti‐CD38 CAR‐T cells without tuning for tumor specificity show not only strong anti‐MM effects but also off‐tumor effects against normal hematopoietic cells.^[^ ^9^ ^]^ Thus, T cell therapies involving CD38 could benefit from avidity engineering.

Recent studies have demonstrated that functional readouts using an autocrine single‐cell format, a paracrine cell–cell interaction format, or a microfluidic mini‐ecosystem format can be used to select highly potent and functional antibodies.^[^ ^12^, ^18^ ^]^ Key to the success of the system reported here was a paracrine cell–cell interaction readout that is fast and has minimal noise interference. CAR‐T cells function by imitating the T cell receptor (TCR). Antigen recognition by the TCR occurs when the TCR binds to antigen on the surface of antigen presenting cells (APC), which then transmit the transmembrane signal and activate T‐cell functions. Like the TCR, Notch is also a membrane receptor specific for paracrine cell–cell signaling. The Notch receptor has been shown to be *trans*‐activated by its cognate neighboring cell surface ligand. The cellular interaction leads to conformation changes in the Notch receptor that renders an otherwise buried intracellular cleavage site (S2) accessible to metalloproteases of the ADAM/TACE family.^[^ ^19^ ^]^ Cleavage at the S2 site generates a membrane‐tethered form of Notch that is further cleaved by the *γ*‐secretase complex, and finally releases the intracellular signal domain.^[^ ^20^ ^]^ Lim and co‐workers developed a surrogate cellular model, synNotch, for the study of T cell activation, in which an engineered Notch receptor, whose intracellular domain contains a transcriptional regulator that is released from the membrane when engagement of cognate extracellular ligand induces intramembrane proteolysis.^[^ ^21^ ^]^ Both the extracellular sensing and the intracellular transcriptional domains of synNotch are modular and can be replaced with heterologous protein domains for customized cell–cell signaling.^[^ ^22^ ^]^ Compared to CAR signaling, which is limited to certain cell types because of defined intracellular signaling domains, e.g., CD3‐*ζ*, CD28, and 4‐1BB, synNotch uses a customized orthogonal signaling machinery that allows signaling outputs from a variety of cell types.^[^ ^23^ ^]^

In this study, we carried out the avidity‐based selection of anti‐CD38 CARs using the combinatorial cellular library of CARs (CCC) approach. We designed and established a paracrine cell–cell panning system using a coculture that consists of one cell population overexpressing the CD38 antigen (donor cells) and a second cell population consisting of a novel gene construct of a synNotch signaling complex and a sub‐library of 10^7^ scFv combinatorial antibodies generated by two rounds of phage enrichment (recipient cells). Normal peripheral blood mononuclear cells (PBMCs) expressing CD38 at physiological levels were used in a negative round of panning of CCC prior to CD38‐targeted enrichment. The anti‐CD38 CAR‐T cells thus selected displayed robust antitumor efficacy both in vitro and in vivo, with minimal on‐target off‐tumor effects. Importantly, this method more efficiently taps into the diversity of the immune system, which in the end can facilitate the discovery of tighter‐binding CARs resulting in less off‐target side effects.

## 2 Results

### 2.1 Construction of Combinatorial Cellular Library of CARs

**Figure 1:**
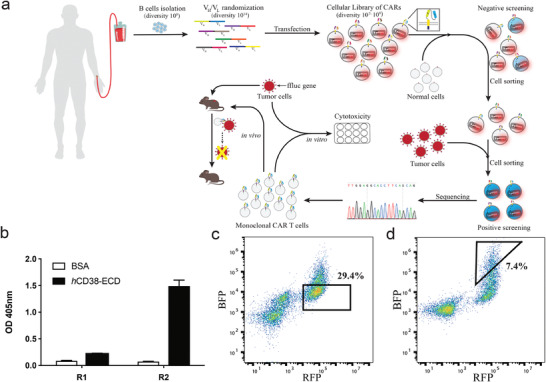
CAR selection from a combinatorial cellular library of CARs (CCC). a) Schematic workflow of avidity‐based screening from CCC. CARs were generated by fusing V_H_ and V_L_ domains. These were then used to replace the extracellular domain of the synNotch receptor system. To identify CAR‐displaying cells susceptible to on‐taraget off‐tumor activation, we carried out a negative round of panning against healthy cells. Cells activated in this negative screen were isolated by cell sorting and discarded. The remaining inactivated cells were then mixed with target tumor cells for the positive screen. The tumor cell activated CAR cells were collected by cell sorting, and the CAR gene in activated cells was sequenced. The most highly enriched CAR genes were selected as candidates and submitted for in vitro and in vivo validations. b) Enrichment of the scFv phage library. The scFv phage library was selected against the *h*CD38‐ECD. Optical density readouts at 405 nm for the *h*CD38‐ECD (black) and a negative control antigen BSA (white) were evaluated. The readout increased significantly after two rounds of enrichment. Data are represented as the mean ± s.e.m. of *n* = 3 technical triplicates. c) Representative FACS plot for negative screen. The CAR‐cell library was cocultured with normal cells expressing CD38 at a low level (*h*PBMC). Activated cells were discarded. The remaining inactivated cells were collected for positive screen. d) Representative FACS plot for positive screen. Inactivated anti‐CD38 CAR expressing cells from (c) were cocultured with CD38 overexpressing cells (K562‐CD38).The enriched activated CAR‐CD38 expressing cells were collected for sequencing.

**Figure** **1**a illustrates the differential selection protocol for tumor‐specific anti‐CD38 CAR‐T cells. We combined the scFv library with the synNotch cell reporting system to select antibodies for the construction of CAR‐T cells. To obtain CAR‐T cells which could be activated by tumor cells with antigen overexpression, but not by normal cells with low antigen expression, an early step in the protocol was to remove candidates that bound to normal cells.

Beginning with a scFv phage library of 10^11^ members, a sub‐library of 10^6^ members was established by panning the original scFv phage library against the purified recombinant antigen protein. The resulting scFv sequences of the sub‐library were cloned and coupled with a synNotch signaling complex using a lentivirus vector, which was then used to transfect mammalian cells. Fluorescence‐activated cell sorting (FACS) sorting was used to isolate the desired cell population.

For proof‐of‐concept, the high‐affinity anti‐CD38 antibody 028 (described in patent WO2011154453) was cloned into a synNotch signaling complex coupled to a lentivirus vector. The transfected 028‐CAR expressing cells were sorted by FACS and then spiked into a sub‐library of cells transfected with the same syn‐Notch‐lentivirus plasmids containing 10^6^ members of scFv sequences (Figure S1a–c, Supporting Information). We carried out a pilot experiment using the antigen presenting K562‐CD38 cells to stimulate CCC at different spiking ratios of 1:10^3^, 1:10^4^, and 1:10^5^. The 1:10^4^ ratio afforded the optimal enrichment of spiked 028‐CAR. Cell sorting using the blue BFP fluorescence signal downstream of the syn‐Notch signaling complex was used to identify cells that recognize the antigen presenting cells. As avidity is influenced both by cell size and density of receptors on the cell surface, we compared the receptor density of HEK293F and primary T cells. As shown in Figure S2 (Supporting Information), both cells displayed similar amounts of synNotch receptors per square micrometer. As mentioned previously, our aim was to select antibodies capable of discriminating between tumor cells with a high level of CD38 expression and normal healthy cells with a low level of CD38 expression. To accomplish this, after transfection into HEK293F cells, the resulting CCC library was screened using two protocols—either directly against a CD38‐overexpressing K562 cell line (K562‐CD38), or by a two‐step process in which it was first prescreened against normal PBMCs to remove any library members binding to cells with low CD38 expression, and then against the K562‐CD38 cell line. Consistent with the literature report that, despite the different levels of CD38 expression, antibody 028 could not distinguish tumor cells from normal cells_._^[^ ^9^ ^]^ Direct screening of the CCC library against K562‐CD38 cells resulted in an enrichment‐fold of 3300 (64/192 prevalence in the output pool) for the sequence encoding 028, whereas the enrichment‐fold was only 260 (5/193 prevalence in the output pool) when the CCC library was prescreened against PBMCs. The significantly reduced enrichment of 028 with prescreening indicates that PBMCs could serve as an efficient negative selection benchmark in vitro. The 028‐encoded CARs apparently recognized both PBMC and K562. We also observed enrichment of new scFv sequences in addition to 028 in both screening formats, suggesting the existence of selective CARs that recognize a specific expression range of CD38. Two CCC sequences were identified and showed enrichment‐folds of 360 and 310, respectively, in the positive but not the negative‐positive screening.

### 2.2 Avidity‐Based Screening of Surface Antigen‐Specific Anti‐CD38 CARs

Encouraged by the above pilot study, we carried out the avidity‐based screening specifically targeting tumor cells that overexpress CD38. As shown in Figure 1a, a scFv phage library of 10^11^ was panned against the recombinant extracellular domain (ECD) of human CD38 (*h*CD38‐ECD). After two rounds of affinity enrichment, the corresponding ELISA signal for the *h*CD38‐ECD group was increased significantly compared to that of the control group (bovine serum albumin, BSA) indicating an enrichment of CD38‐specific antibodies (Figure 1b). The DNA sequences obtained from positive scFv clones (10^6^) were subcloned into a syn‐Notch‐lentivirus vector and transfected into 10^7^ HEK293F cells to generate a CD38‐focused CCC library. Then, for avidity‐based screening, the CCC library was first cocultured with normal human PBMCs, which have a low level of CD38 expression. Activation of library members by PBMCs was indicated by positive BFP fluorescence, and these activated cells were filtered and removed via cell sorting (Figure 1c). The remaining cells were collected and cocultured with K562‐CD38 cells which overexpress CD38. Activated cells showing positive fluorescence of BFP were collected by cell sorting (Figure 1d), and lysed for PCR amplification of the encoded scFv sequences. After sequencing and gene clustering, scFv sequences with the highest enrichment were selected, and confirmed individually using the synNotch cell–cell signaling system. Of the five tested scFv sequences, two showed significant activation of synNotch signaling (Figure S1d–g, Supporting Information).

### 2.3 Binding Affinity of scFv Antibodies RP02 and RP03

**Figure 2:**
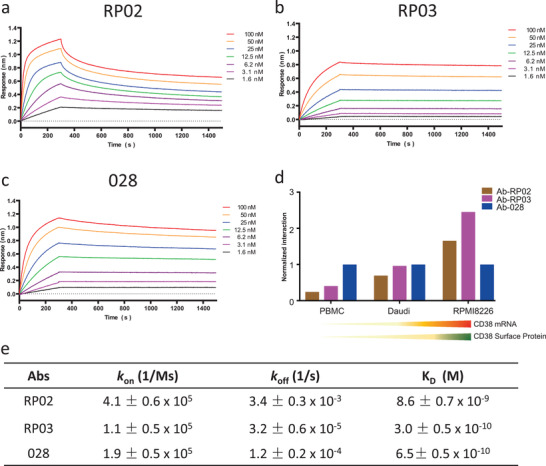
Binding affinity of anti‐CD38 scFv antibodies. a–c) Binding kinetics of anti‐CD38 RP02, RP03, and 028 scFv antibodies against purified *h*CD38‐ECD were investigated by biolayer interferometry. The dissociation constants were 8.6, 0.3, and 0.65 × 10^−9^ m for RP02, RP03, and 028, respectively. d) Purified scFv antibodies selectively bind to tumor cells with different CD38 expression levels. ScFv antibodies were incubated with *h*PBMC, Daudi, or RPMI8226 cells and stained with fluorescence labeled secondary antibody. The mean fluorescence intensity (MFI) of each antibody with different cells was normalized to the MFI of 028 scFv antibody with the corresponding cells. The mRNA and cell surface expression levels of CD38 are indicated below; the detailed CD38 expression analyses are shown in Figure S4 (Supporting Information). e) Kinetic rates and binding constants were obtained by fitting the binding curves generated for individual curves to the 1:1 interaction model.

Binding affinity is a key biophysical feature for antibody targeting. We used surface plasmon resonance to evaluate the binding affinity of the two anti‐CD38 combinatorial scFv antibodies (RP02 and RP03) identified from the avidity‐selection protocol. Interactions between the purified scFv antibodies and the *h*CD38‐ECD/*m*CD38‐ ECD proteins were first measured using the surface plasma resonance (SPR) method on an Octet. Both RP02 and RP03 displayed potent binding interactions (*K*_D_ = 8.6 and 0.3 × 10^−9^ m, respectively), but highly different kinetics with respect to the extracellular domain of human CD38 (**Figure** **2**a–c,e). The binding of RP02 showed fast *k*_on_ and fast *k*_off_ kinetics, whereas the binding of RP03 and 028 showed fast *k*_on_ but slow *k*_off_. In addition, dissociation of RP02 displayed an apparent biphasic kinetics. Biphasic dissociation was also observed for other antibodies.^[^ ^24^ ^]^ The interactions appeared to be species specific. Despite over 57% sequence identity between *h*CD38‐ECD and *m*CD38‐ECD, both RP02 and RP03 showed no cross‐species interations with the ECD domain of mouse CD38 (Figure S3, Supporting Information).

To determine the interaction between scFv antibodies and cell surfaces displaying CD38, three cell lines with high to low CD38 expression, RPMI8226 > Daudi > PBMC, were selected as the target cells. It was noted that the avidity‐selected RP02 and RP03 recognized the target cells in a CD38 expression‐dependent manner (Figure 2d). Compared to 028, both RP02 and RP03 showed stronger interactions with high CD38 expressing cells, RPMI8226. By contrast, their interactions with low CD38 expressing cells such as Daudi and PBMC were similar or weaker than that seen with 028.

### 2.4 CD38‐Dependent Cytokine Release by RP02 CAR‐T Cells

**Figure 3:**
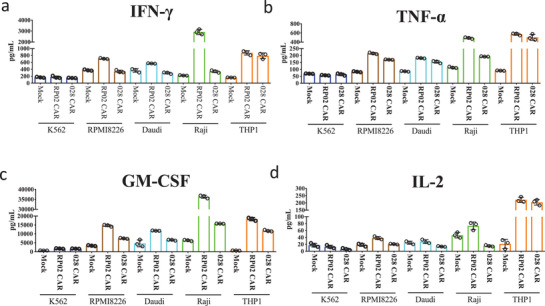
Cytokine secretion of anti‐CD38 RP02 and 028 CAR‐T cells. CAR‐T cells were coincubated with the CD38+ cell lines, i.e., RPMI8226, Daudi, Raji, THP1, or CD38− cell line K562 at E:T ratio of 4:1 for 24 h. Cytokine secretion by anti‐CD38 RP02 and 028 CAR‐T cells was measured using the AlphaLISA assay. a–d) Graph shows the secretion of IFN‐*γ*, TNF‐*α*, GM‐CSF, and IL‐2. Data are represented as mean ± s.e.m. of *n* = 3 technical replicates.

To demonstrate activation of RP02 CAR‐T cells, cytokine secretion stimulated by CD38‐positive tumor cell lines was measured. The level of cytokine production, including interferon *γ* (IFN‐*γ*), interleukin‐2 (IL‐2), tumor necrosis factor *α* (TNF‐*α*), and GM‐CSF, appeared to correlate with the level of CD38 expression in the tumor cells, i.e., Raji, Daudi, RPMI8226, and THP1 cells stimulated more cytokine production than K562 cells (**Figure** **3** and Figure S4, Supporting Information). Although the binding of RP02 antibody to CD38 is weaker than that of 028 antibody, when activated by CD38‐positive tumor cell lines, RP02 CAR‐T cells consistently produced a stronger cytokine response than 028 CAR‐T cells.

### 2.5 Tumor Cell Killing by Multiple Constructs of CD38 CAR‐T Cells

**Figure 4:**
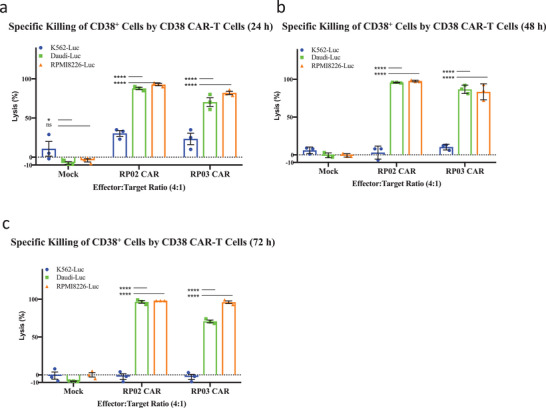
Tumor cell lytic capacity of anti‐CD38 RP02 and RP03 CAR‐T cells. a–c) Anti‐CD38 CAR‐T cells generated significantly stronger cytotoxicity for CD38+ target tumor cells (Daudi and RPMI8226) in comparison to mock T cells after coculturing for different incubation times, e.g., 24, 48, and 72 h at an effector:target (E:T) ratio of 4:1. Data are represented as the mean ± s.e.m. of *n* = 3 technical replicates. Significance was considered as *p < 0.05; **p < 0.01; ***p < 0.001; ****p < 0.0001.

**Figure 5:**
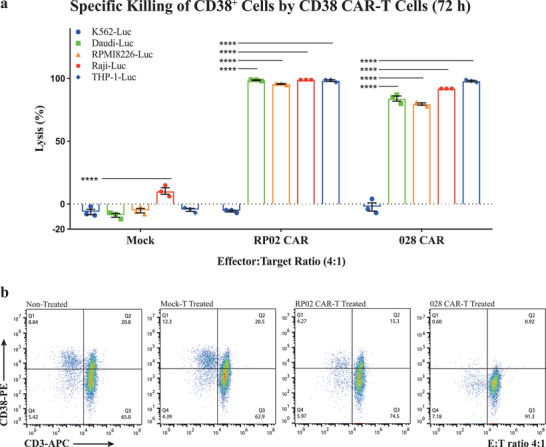
Evaluation of antitumor activity and safety of anti‐CD38 RP02 and 028 CAR‐T cells. a) Specific killing of various CD38^+^ tumor cells by RP02 and 028 CAR‐T cells after coculture for 72 h at an E:T ratio of 4:1. The luciferase signals generated by surviving malignant cells were recorded to evaluate the antitumor activity. Data are represented as mean ± s.e.m. of *n* = 3 technical replicates. b) Specific killing of PBMC cells by RP02 and 028 CAR‐T cells. PBMC cells were cocultured with CAR‐T cells for 72 h at an E:T ratio of 4:1. The population of CD38+ PBMC cell lysis was used to evaluate the off‐tumor activity of CAR‐T cells. Significance was considered as *p < 0.05; **p < 0.01; ***p < 0.001; ****p < 0.0001.

The antitumor function of CAR‐T cells is, of course, of primary importance in evaluating their therapeutic efficacy. We determined the lytic capacity of RP02 and RP03 CAR‐T cells versus two CD38‐positive tumor cell lines (Daudi and RPMI‐8226). Initially, multiple cell lines were examined using qPCR and FACS to quantify their level of CD38 expression (Figure S4, Supporting Information). The K562 cell line was chosen as the antigen‐negative control, because neither CD38 mRNA nor cell surface CD38 was detected in the quantification. The high‐affinity antibody 028 was used to generate functional CD38 CAR‐T cells as the positive control.^[^ ^14^ ^]^ For the tumor cell lysis assay, luciferase genes were transduced into the tumor cell lines to form reporter cells. These were then incubated with CAR‐T cells using two effector to target ratios (E:T, 4:1 and 1:1) and three incubation times (24, 48, and 72 h). The effect of cell lysis was determined using the luciferase signal produced by surviving malignant cells. Both RP02 and RP03 CAR‐T cells from avidity‐based selection were capable of lysing the tumor cell lines of Daudi and RPMI‐8226. Almost all tumor cells were lysed in 48 h with an E:T ratio of 4:1, whereas most of the K562 cells survived (**Figure** **4**), indicating that the cytotoxic function of the CAR‐T cells is CD38‐specific. Interestingly, we did observe some differences in the efficiency of tumor cell lysis. At an E:T ratio of 1:1, both RP02 and RP03 CAR‐T cells lysed over 80% of Daudi cells in the first 24 h, but under the same conditions only 40% of RPMI‐8226 cells were lysed, indicating some degree of tumor cell selectivity (Figure S5, Supporting Information). Of note is that, despite the fact that RP02 has a weaker binding affinity to purified CD38, the RP02 CAR‐T cells were more potent than RP03 and 028 CAR‐T cells in terms of cytotoxicity for all tumor cell lines tested, including Daudi, RPMI8226, Raji, and THP cells (**Figure** **5**a).

### 2.6 Tumor Selective Effect of RP02 CAR‐T Cells

Our aim was to generate functional CAR‐T cells with minimal off‐tumor on‐target effects. To evaluate this characteristic in our anti‐CD38 CAR‐T cell constructs, we examined their level of cytotoxicity toward tumor cells overexpressing CD38 as compared to normal cells with physiological levels of CD38 expression. CD38 is widely expressed. We collected cells originating from a number of different tissues, including Daudi (peripheral blood), RPMI8226 (peripheral blood), Raji (lymphoblast), THP‐1 (monocyte), K562 (bone marrow), A549 (lung), PC3 (prostate), and primary PBMC. Expression of CD38 on these cells was determined using qPCR and flow cytometry (Figure S4, Supporting Information). The mean fluorescence intensity (MFI) reflecting the cell surface CD38 expression level on different cell types was ranked in decreasing order as follows: RPMI 8226, Raji, Daudi, THP1, PBMC, K562, A549, and PC3. For a side‐by‐side comparison of the on‐tumor and off‐tumor effects, the tumor target cells were transfected with the luciferase reporter gene. After incubation of CAR‐T cells with selected target cells, we determined the lysis of target cells via a luciferase reporter assay. The tested CAR‐T cells lysed target cells in a CD38‐expression‐dependent manner (Figure 5a). As could be anticipated, the 028 CAR‐T cells lysed all cells expressing CD38, including the healthy PBMC cells (Figure 5b). In contrast, the RP02 CAR‐T cells selectively lysed those cells which overexpressed CD38 (i.e., RPMI‐8226, Raji, Daudi, and THP1). It was noted that a certain amount of CD38 positive cells (13.2%) in PBMC were lysed by RP02 CAR‐T cells (Figure 5b). Interestingly, the percentage of apparent killing of PBMC coincides with the percentage of residual enrichment of 028‐CAR (7.8%), which may indicate that a systemic deviation exists in the paracrine cell–cell assay system. This indicates that when constructing CAR‐T cells, including a negative‐selection step in which CARs binding to cells with low level antigen expression are removed, can indeed result in CAR‐T cells with minimal off‐tumor effects.

**Figure 6:**
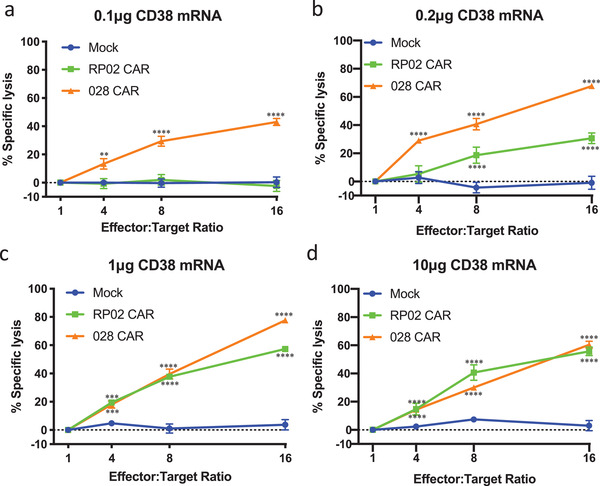
CD38 expression‐dependent activity of CAR‐T cells. a–d) K562 cells were electroporated with CD38 mRNA ranging from 0.1, 0.2, 1, and 10 µg as indicated. 1 d after the electroporation, CAR‐T cells were cocultured with electroporated K562 cells at different E:T ratios. After 8 h coculture, the percentage of specific lysis was calculated. All groups were compared with Mock‐T cells. Data are represented as mean ± s.e.m. of *n* = 3 technical replicates. Significance was considered as *p < 0.05; **p < 0.01; ***p < 0.001; ****p < 0.0001.

Additional experiments support the conclusion that RP02 CAR‐T cells have been engineered to discriminate between cells expressing high and low levels of CD38. Antigen dose‐dependent reactivity was confirmed using a luciferase‐based cytolytic T‐cell (CTL) assay. Untreated K562 cells are not lysed by RP02 CAR‐T cells, but when K562 target cells were transfected with 1 or 10 µg CD38 mRNA, they were effectively lysed by RP02 CAR‐T cells as well as by 028 CAR‐T cells (**Figure** **6**). For the target cells transfected with 0.2 µg CD38 mRNA, on the other hand, the affinity‐selected 028 CAR‐T cells exhibited more potent lytic activity than the avidity‐selected RP02 CAR‐T cells. Finally, after electroporation with 0.1 µg CD38 mRNA, which leads to a CD38 expression level similar to that of PBMCs, only 028 CAR‐T cells were able to kill target cells (Figure 6a). Again, indicating that RP02 CAR‐T cells, while cytotoxic for CD38‐expressing tumor cells, show very little activity toward cells expressing the low levels of CD38 that would be found in normal tissue.

### 2.7 Peptide Mapping of RP02 and 028 Binding Sites on CD38

**Figure 7:**
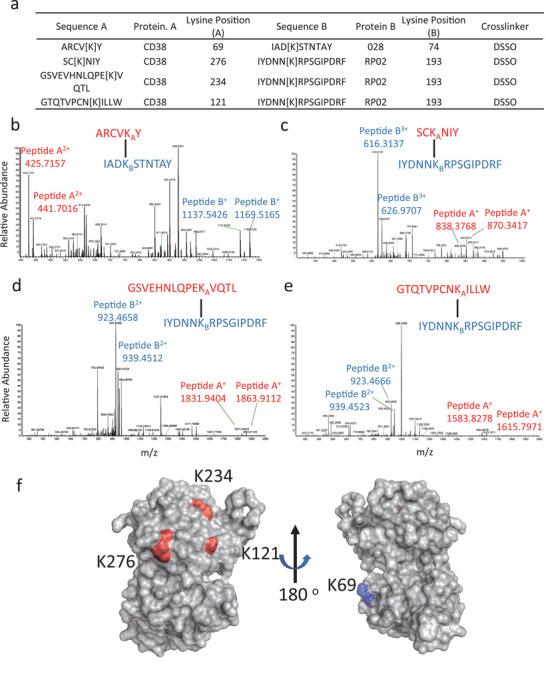
Identification of RP02 and 028 binding site on the *h*CD38‐ECD. a) *h*CD38‐ECD was crosslinked with RP02 or 028 scFv using DSSO. Crosslinked peptides were identified by high‐resolution MS analyses. b–e) Secondary MS spectra identified crosslinked peptides ARCVKY‐IADKSTNTAY, SCKNIY‐IYDNNKRPSGIPDRF, GSVEHNLQPEKVQTL‐ IYDNNKRPSGIPDRF, and GTQTVPCNKILLW‐ IYDNNKRPSGIPDRF. Tertiary MS data are shown in Figures S6–S9 (Supporting Information). The MS data were deposited in the proteomics identifications (PRIDE) database with accession number PXD019713. f) Location of RP02 and 028 binding sites on *h*CD38‐ECD. The lysine crosslinked with 028 (K69) is shown in blue and the lysine crosslinked with RP02 (K121, K234, and K276) is shown in red. The structure of *h*CD38‐ECD (PDB 1yh3) is displayed by PyMOL.

To dissect the mechanism of interaction between RP02 or 028 with CD38, peptide mapping experiments using high‐resolution mass spectrometry (MS) were carried out. The binary complex of *h*CD38‐ECD with RP02 or 028 was first crosslinked by the collision‐induced dissociation (CID)‐cleavable crosslinker disuccinimido sulfoxide (DSSO),^[^ ^25^ ^]^ and then digested using chymotrypsin. Integrated analyses of CID‐induced cleavage of interlinked peptides in MS^2^ and MS^3^ of single peptide chain fragment ions revealed distinct binding peptides of *h*CD38‐ECD for RP02 and 028 (**Figure** **7**a). 028 appeared to bind at the N terminus of *h*CD38‐ECD near lysine 69 (K69) (Figure 7b,f and Figure S6, Supporting Information), whereas RP02 interacted at a region close to three adjacent lysine residues (K121, K234, and K276) (Figure 7c–f and Figures S7–S9, Supporting Information).

### 2.8 In Vivo Antitumor Effect of RP02 CAR‐T Cells

**Figure 8:**
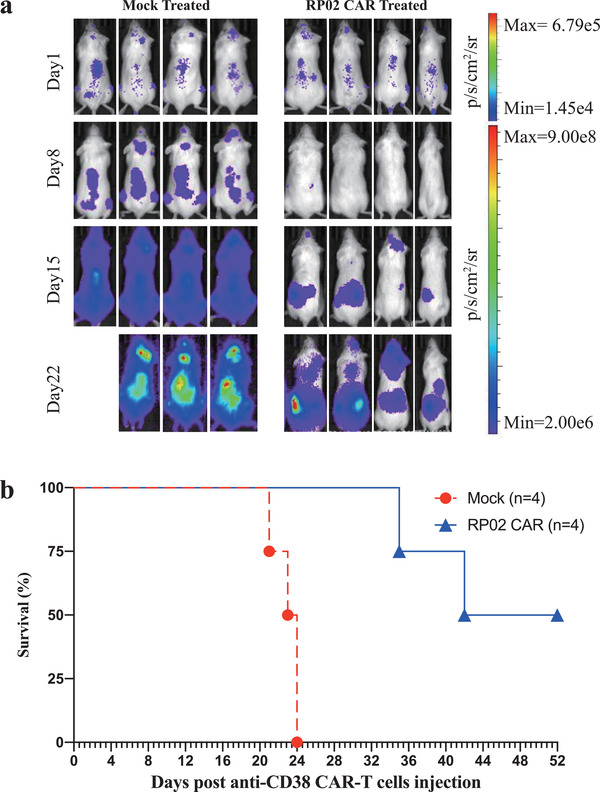
Anti‐CD38 CAR‐T cell‐mediated antitumor response in xenografted immunocompromised mice. a) NOG mice were intravenously implanted with luciferase‐transduced human CD38+ Daudi cells (1 × 10^6^ cells). Upon successful engraftment, mice were intravenously treated with 1 × 10^7^ mock‐T or RP02 CAR‐T cells once. Tumor load was quantified by BLI measurements. Bioluminescent images are shown per group at weekly intervals. b) Survival curves of mice receiving mock‐T or RP02 CAR‐T cells (*n* = 4).

We then evaluated the in vivo antitumor effects of RP02 CAR‐T cells. We used the luciferase‐transduced Daudi tumor cells to generate the xenograft murine model. As illustrated in **Figure** **8**a, in the control group treated with mock T cells, tumors showed a fast progression. Although not curative, treatment of the tumor‐bearing mice with RP02 CAR‐T cells induced a significant antitumor effect and longer overall survival (Figure 8b).

## 3 Conclusion

The biggest challenge in large‐scale application of CAR‐T technology to cancer therapy is that many target antigens are TAAs rather than tumor‐specific antigens (TSAs), and have a broad tissue distribution in the body. Even a low level of target antigen expression on normal cells can lead to on‐target off‐tumor effects resulting in unacceptable toxicity. In an attempt to overcome this difficulty, we took advantage of the possibility that differential expression of TAAs on normal versus tumor cells may lead to differences in the local surrounding environment and unique epitope targeting regions. We hypothesized that selecting CARs based on avidity binding to such a specific membrane TAA epitope region would allow the construction of CAR‐T cells capable of discriminating between tumor and normal cells, even when both express the cognate TAA. Herein, we describe a combinatorial library approach in which selection is based on avidity, rather than simple recognition. Using as an example the TAA CD38, we showed that avidity‐specific CARs can be used to construct CAR‐T cells capable of selectively targeting CD38‐expressing tumor versus normal cells.

Previous studies found that affinity optimization of tumor antigen binding resulted in a CAR with improved selectivity to high tumor antigen density cells and reduced the risk of on‐target off‐tumor effects.^[^ ^9^, ^10^, ^26^ ^]^ This improved therapeutic index was achieved mainly by tuning down the affinity between the scFv antibody and its cognate target antigen sequence, a process which appeared to have a more profound effect when the level of membrane antigen expression was low. Affinity tuning using purified antigen sequences does not take into account the spatial arrangement of antigen on the cell membrane. As a mitigation strategy, the approach could be at the expense of increasing off‐target risk. The tissue‐selective anti‐CD38 CARs identified here using our combinatorial library approach have different primary sequences, especially in the CDR3 region, compared to the known tissue nonselective antibody (Figure S10, Supporting Information). We feel this likely indicates a new epitope recognition region on the membrane‐expressed CD38. Crosslinking experiments with RP02 mapped the interaction region of the corresponding scFv antibodies to the top of *h*CD38‐ECD parallel to the cell membrane. This orientation is different from that seen with antibody 028 (Figure 7f). From the affinity measurement, RP02 bound to the purified CD38 with an apparent *K*_D_ value of 8.6 × 10^−9^ m and displayed a biphasic dissociation kinetics, much weaker than the *K*_D_ value of the known scFv antibody 028 (0.65 × 10^−9^ m) (Figure 2a,c), yet both showed similar efficacy in terms of tumor cell lysis (Figure 5a). Similarly, Chmielewski et al. described an optimal affinity in the range of 10^−8^ m for tumor‐selective ErbB2 CAR‐T cells,^[^ ^27^ ^]^ whereas Liu et al. showed 10^−9^ m for the same targeting antigen.^[^ ^10^ ^]^ Both CARs demonstrated impressive efficacies with minimal on‐target off‐tumor effects. Thus, cellular avidity appears dependent not only on the cognate interactions of epitope sequences but also on the spatial arrangement of surface antigens, which is closely associated with the membrane expression level.^[^ ^28^ ^]^ As a cell‐surface molecule, CD38 is highly conserved in phylogeny and functionally pleiotropic, acting simultaneously as an ectoenzyme and a membrane receptor.^[^ ^15^ ^]^ It has been suggested that CD38 participates in the immunologic synapse (IS) formation during the process of antigen presentation and T cell activation.^[^ ^29^ ^]^ The mRNA‐dependent RP02 CAR did not interfere with the intrinsic CD38 function in normal PBMC/T cells (Figure 5b). The RP02 CAR appeared to establish the IS contact with antigen‐presenting cells only when its CD38 mRNA level was above a threshold (Figure 6). The high specificity of the RP02 CAR endows RP02 CAR‐T cells with a functionally increased level of T cell activity, as demonstrated by higher levels of cytokine secretion (Figure 3) and maximal killing of multiple tumor cells (Figure 5a) when compared to 028 CAR‐T cells.

The key to construction of an effective CCC is a coculture system that is capable of using orthogonal transcription factors and responds independently without disturbing signaling of host cells. In our system, both T cell activation and synNotch signaling can be elicited by antigen‐presenting cells, but in principle, with customized signaling any host cell could be used. We showed the receptor densities of CARs on the cell surface were similar for T cells and HEK293F, despite HEK293F cells being much larger (Figure S2, Supporting Information). SynNotch signaling has been successfully applied in various CAR‐T therapies against different TAAs, such as CD19, Axl, Apj, and ROR1.^[^ ^22^, ^30^ ^]^ Our current study demonstrated that a combinatorial single chain antibody (scFv) library could be displayed on the cell surface and screened using synNotch‐mediated cell–cell interactions. After initial affinity enrichment, the cellular scFv library provided a pool of at least 10^6^ possible CARs recognizing the same target surface antigen. This population was further enriched via avidity‐based selection to remove members that recognized normal cells with endogenous levels of target antigen expression, thereby leaving only CARs with the ability to discriminate between cells with high and low levels of target antigen expression.

In summary, our results demonstrate that using a combinatorial library approach together with a synNotch‐based cell–cell interaction selection system could be a promising strategy to select and optimize CARs for construction of CAR‐T cells having both safety and efficacy. The anti‐CD38 CAR‐T cells developed using this approach retained potent antitumor activity and showed very low toxicity toward normal cells with a physiological level of CD38 expression. This approach used cellular activation rather than affinity as readout, and would have a wide range of applications, such as multi‐antigen activation, epitope optimization, CAR logic design—all of which could be used to improve the safety and efficacy of cell‐based therapy. More importantly, the combinatorial library approach makes it possible to generate a polyclonal population of CAR‐T cells that specifically targets disease cells. By following a similar clinical procedure of CAR‐T treatment, “synthetic immunity” could be eventually applied to substitute for a failed “natural immunity” of an individual. Thus, the CCC approach opens the door for immune cell therapy to become a general practice, and eventually, point‐of‐care in clinical settings. This highly personalized therapeutic approach, nevertheless, will face new challenges and calls for a set of new standards and regulations, for example, in good manufacturing practice (GMP), quality and safety controls. And as with other immune therapies, success will track with the ability to capture diversity.

## 4 Experimental Section

##### Combinatorial Antibody Library Enrichment Targeting hCD38‐ECD

Recombinant human CD38 extracellular domain (amino acids 43–300, *h*CD38‐ECD) (Sino Biological, Cat. No. 10818‐H08H) was biotinylated with EZ‐Link NHS‐PEG_4_‐biotin and biotinylation kits (Thermo Scientific, Cat. No. 21455). In general, 300 µL of a combinatorial scFv phage library (10^11^) was first incubated with 2.5 µg biotinylated *h*CD38‐ECD. The resulting *h*CD38‐ECD phagemids were pulled down using 200 µL streptavidin‐coated Dynabeads M‐280 (Life Technologies, Cat. No. 11205), and the unbound phagemids were removed by washing three times with phosphate buffered saline (PBS) (HyClone, Cat. No. SH30256.01) and PBST (PBS supplemented with 0.1% Tween‐20), respectively. The bound phagemids were then eluted using a glycine‐HCl buffer (pH 2.2). XL‐1 blue bacteria infected with the eluted phagemids were grown at 37 °C on LB agar plates supplemented with ampicillin (0.1 mg mL^−1^) overnight. Phage were scraped from the plate and amplified for the next round of panning with a helper phage VCSM13.

##### Construction of Avidity‐Based Combinatorial Cellular Library

The lentiviral synNotch library was established by fusing the *h*CD38‐ECD enriched combinatorial scFv library described above with the lentiviral synNotch vector, which is a gift from Wendell Lim (Addgene plasmid no. 79125; http://n2t.net/addgene:79125; RRID:Addgene_79125).^[^ ^21^ ^]^ As shown in Figure S1h (Supporting Information), the synNotch construct constitutes a N‐terminal extracellular domain containing a *h*CD8*α* signaling peptide (MALPVTALLLPLALLLHAARP), a myc peptide (EQKLISEEDL), and a CAR; a transmembrane domain consisting of *m*Notch1 core (from 1427 to 1752 amino acids); and an intracellular domain consisting of Gal4‐VP64. The effector construct (Figure S1i, Supporting Information) was designed to monitor chimeric Notch signaling, in which synNotch activation resulted in Gal4‐VP64 release and, subsequently, BFP expression through interaction between the released Gal4‐VP64 and Gal4 DNA binding domain. A *m*Cherry fluorescent protein was used in the construct as an internal transduction marker that could be monitored using the RFP channel in FACS. Lentivirus was produced by cotransfecting 1.5 µg synNotch library plasmids, 0.6 µg lentivirus envelope plasmid pMD2G, and 0.9 µg packaging plasmid psPAX2 with 3 × 10^6^ HEK293‐T cells plated in six‐well plates using Lipofectamine 3000 (Thermo Fisher, Cat. No. L3000015) transfection reagent. Viral supernatants were collected 60 h after transfection, and filtered with a 45 µm pore filter. The titer of lentivirus was determined using a Lenti‐X p24 rapid titer kit (Clontech, Cat. No. 632200). The virus was aliquoted and kept at −80 °C for long‐term storage.

##### Selection of Functional CARs from the Combinatorial Cellular Library

HEK293F cells were infected with the lentiviral synNotch library at a multiplicity of infection (MOI) of 0.6. After 8 h cultivation, cells were exchanged into a fresh medium without virus, and incubated for an additional 48 h at 37 °C in the presence of 5% CO_2_. To select antibodies capable of discriminating tumor cells with a high level of CD38 expression versus normal healthy cells with a low level of CD38 expression, the CCC library was screened using a two‐step panning protocol, in which a prescreening against normal PBMCs was followed by panning against the K562‐CD38 cell line which overexpresses CD38. The coculture of CAR‐expressing cells with antigen‐presenting tumor cell lines or normal PBMCs was cultivated at a ratio of 1:2 at 37 °C for 24 h. Negative selection using normal PBMCs was performed by sorting the RFP^+^BFP^−^ population on FACS. The resulting cells were cultured in a fresh media, and cocultured with K562‐CD38 cells at a ratio of 1:2 at 37 °C for 24 h. The activated cells showing positive fluorescence of BFP were collected by FACS, and subjected to gene extraction. For the one‐step positive selection, cells transduced with CCC were cocultured with K562‐CD38 cells at a ratio of 1:2 at 37 °C for 24 h. The activated cells showing positive fluorescence of BFP were collected by cell‐sorting and subjected to gene extraction. The CAR genes were extracted from sorted cells by DNeasy Blood and Tissue kit (Qiagen, Cat. No. 69504) and subcloned in a lentiviral synNotch vector. The resulting vector plasmids were transfected into a TransStbl3 competent cell (TransGen, Cat. No. CD521). To estimate the enrichment of CAR sequences, ≈200 single colonies were randomly picked for Sanger sequencing. For each unique sequence (X), the counts were first normalized versus the total counts. The normalized input and output counts were then used to calculate the enrichment fold as shown in the following

(1) $$\begin{array}{ccc} & & \mathrm{Enrichmen}t_{X}=\;\mathrm{NormalizedCoun}t_{X,\;\mathrm{output}}/\mathrm{NormalizedCoun}t_{X,\;\mathrm{input}} \end{array}$$

The percentage of residual enrichment of 028‐CAR was calculated as follows

(2) $$\begin{array}{ccc} & & \%\mathrm{Enrichment}\;\\ & & \;=\;\mathrm{Enrichmen}t_{\mathrm{negative}-\mathrm{positive}\;\mathrm{selection}}/\mathrm{Enrichmen}t_{\mathrm{positive}\;\mathrm{selection}} \end{array}$$

##### Construction of CAR‐T Cells

Anti‐CD38 CAR‐T cells were generated by nucleofection with a piggyBac transposon system (System Biosciences, Cat. No. PB210PA‐1). The anti‐CD38 CAR piggyback transposon vectors were constructed according to an established protocol.^[^ ^31^ ^]^ A total of 2 × 10^7^ PBMC cells (Hemacare, Cat. No. PB009C‐3) were resuspended in 100 µL of suspension solution from the human T cell Nucleofector kit (Lonza, Cat. No. VPA‐1002) and electroporated with 10 µg anti‐CD38 CAR piggyBac transposon vector and 5 µg Super piggyBac transposase plasmid on a Nucleofector II/2b device (Lonza) following the U‐014 program (designed for unstimulated T cells). 1 d after nucleofection, 50 ng mL^−1^ CD3 antibody (Miltenyi, Cat. No. 130093387) and 300 IU mL^−1^ human IL‐2 (R&D Systems, Cat. No. 202‐GMP‐050) were added to the resulting T cells for stimulation and expansion. As feeder cells to improve the expansion, allogeneic PBMCs from five donors were irradiated at 40 Gy on an X‐ray biological irradiator (Rad Source technologies), and added to the T‐cell culture at a ratio of 10:1. After 10 d, CAR‐transfected and vector‐transfected T cells (mock T cells) were analyzed by flow cytometry on a CytoFLEX (Beckman Coulter). The Myc tag was used as the cellular marker for membrane expression of the CAR. CAR‐T cells were stained with phycoerythrin (PE)‐conjugated mouse anti‐Myc tag antibody (Cell signaling, Cat. No. 3739S) and allophycocyanin‐H7 (APC‐H7)‐conjugated mouse anti‐human CD3 (BD pharmingen, Cat. No. 560275) for 60 min at room temperature. After washing three times using FACS buffer (PBS+1% BSA), the stained cells were measured by flow cytometry on a CytoFLEX (Beckman Coulter). The data were analyzed and plotted using FlowJo.

##### Surface Receptor Density Comparison of HEK293F versus T Cells

HEK293F or primary T cells were transfected with the 028‐synNotch lentivirus with a MOI of 0.6. A total of 60 resulting cells were randomly selected and visualized by microscopy (EVOS M5000, Life Technologies). The diameters of these cells were determined using ImageJ. Surface expression of CAR on a T or HEK293F cell was determined by flow cytometry. The cellular CAR expression was analyzed using an anti‐myc primary antibody (Cell Signaling Technology, PE conjugated c‐Myc(D84C12) Rabbit mAb, Cat. No. 14819S) by cellular MFI. The CAR density on the cell surface was normalized by the cell surface area. Data were shown in Figure S2 (Supporting Information).

##### Quantification of CD38 Expression by qPCR

A total of 5 × 10^6^ cells each of the following cells including Daudi, RPMI8226, Raji, THP‐1, Jurkat, PBMC, K562, A549, and PC3 were aspirated into a RLT buffer (QIAGEN, Cat. No. 79216). Total RNA was extracted using the RNeasy Micro Kit (QIAGEN, Cat. No. 74104). cDNA was generated using High Capacity cDNA Reverse Transcription Kit (Applied Biosystems, Cat. No. 00494742). In a 20 µL reaction volume consisting of Power SYBR Green Master Mix (Vazyme, Cat. No. Q711‐02), 5 ng cDNA and 200 × 10^−9^ m gene‐specific forward and reverse primers (primers for CD38: Forward: CAACTCTGTCTTGGCGTCAGT; Reverse: CCCATACACTTTGGCAGTCTACA; primers for GAPDH: Forward: GGAGCGAGATCCCTCCAAAAT; Reverse: GGCTGTTGTCATACTTCTCATGG), gene amplifications were performed for 40 cycles on a CFX96 Real‐Time PCR Detection System (Bio‐Rad). Comparative quantification of the CD38 expression in the cells was performed based on cycle threshold (Ct) normalized to GAPDH.

##### Cytotoxicity Assay

The cytotoxicity of CAR‐T cells was assessed using a luciferase releasing assay. A total of 1 × 10^4^ luciferase reporter gene transfected cells (including Daudi, RPMI8226, Raji, THP‐1, Jurkat, PBMC, K562, A549 .and PC3) were seeded in 96‐well plates, respectively. The cells were cultured in complete RPMI 1640 media with 10% fetal bovine serum (FBS) at 37 °C. Mock T, 028 CAR‐T, and RP02 CAR‐T cells were added at different effector‐to‐target cell ratios as indicated in the figures. The luciferase signals were recorded after 24, 48, and 72 h. 0.75 mg mL^−1^ d‐luciferin K^+^ salt (PerkinElmer; Cat. No. 122799) was added to the cell culture as the substrate for luciferase, and the signals were read immediately using an EnSpire Multimode plate reader (PerkinElmer).

PBMCs were prestained with Cell Trace Blue (Invitrogen, Cat. No. C34568) and cocultured with unstained CAR‐T cells or mock T cells at 37 °C for 72 h. Only the blue fluorescence positive cells (PBMC cells) were collected by FACS for analysis in Figure 5b.

##### Binding Kinetics

The binding kinetics of scFv antibodies with *h*CD38‐ECD were evaluated on an Octet Red96 system (ForteBio) at room temperature. Biotin conjugated *h*CD38‐ECDs were captured on a SA‐coated biosensor (ForteBio). The baseline was recorded for 60 s in a running buffer (PBS, 0.02% Tween‐20, and 0.05% BSA, pH 7.4). Afterward, the sensors were subjected to an association phase for 300 s in wells containing scFv antibodies diluted in the buffer. In the dissociation step, the sensors were immersed in the running buffer for 1200 s. The average *k*_on_, *k*_off_, and *K*_D_ values were calculated from all the binding curves based on the fitting with a 1:1 Langmuir binding model. For RP02, because of its biphasic dissociation kinetics, the *K*_D_ value of RP02 was estimated using the initial 0–330 s to fit into a 1:1 binding model. As the steady state of dissociation was not taken into account, the *k*_off_ and *K*_D_ were underestimated.

##### Cytokine Measurement

1 × 10^5^ K562, Daudi, RPMI8226, Raji, and THP‐1 cells were seeded in 96‐well plates. 4 × 10^5^ Mock T, 028 CAR‐T, or RP02 CAR‐T cells were added and cocultured in 200 µL of RPMI 1640 medium with 10% FBS. After 24 h, the supernatant was collected. The production of cytokines, including interferon (IFN)‐*γ*, tumor necrosis factor (TNF)‐*α*, granulocyte‐macrophage colony‐stimulating factor (GM‐CSF), and IL‐2 was determined using AlphaLISA kits from PerkinElmer (IL‐2, Cat. No. AL221C/F; IFN‐*γ*, Cat. No. AL217C/F; TNF‐*α*, Cat. No. AL208C/F; and GM‐CSF, Cat. No. AL216C/F) according to the manufacturer's instructions.

##### Chemical Crosslinking and Mass Spectrometry

RP02 and 028 antibodies were crosslinked with *h*CD38‐ECD using CID‐cleavable crosslinker DSSO following the described procedure.^[^ ^25^, ^32^ ^]^ The antibody and *h*CD38‐ECD were mixed in PBS and incubated for 30 min on ice. Crosslinking was performed for 30 min by adding DSSO (Thermo Scientific) to the protein/antibody solution with 100 molar excess. The crosslinking reaction was quenched by excess Tris (1 m, pH 8.0). The crosslinked products were digested with chymotrypsin. The LC MS*^n^* data of digested peptides were collected on Orbitrap Fusion Tribrid (Thermo Scientific) with an on‐line NanoLC system and analyzed using CID‐MS^2^‐MS^3^ strategy as previously described.^[^ ^33^ ^]^ Monoisotopic mass of parent ions and corresponding fragment ions, parent ion charge states, and ion intensities from LC MS^2^ and LC MS^3^ spectra were extracted using Xcalibur v 3.0 (Thermo Scientific). Database searching was performed using Proteome Discoverer v 2.2 software (Thermo Scientific). Chymotrypsin was set as the enzyme with two missed cleavages being allowed as the maximum values. Protein N‐terminal acetylation, methionine oxidation (15.995 Da), carbamidomethyl cysteine (57.021 Da), hydrolyzed lysine DSSO (176.014 Da), and lysine DSSO Tris (279.078 Da) were selected as variable modifications. In addition, to account for the residual crosslinker, three defined modifications on uncleaved lysines were chosen, including alkene (C_3_H_2_O, 54 Da), sulfenic acid (C_3_H_4_O_2_S, 104 Da), and thiol (C_3_H_2_SO, 86 Da) modifications. A false discovery rate (FDR) of 1% was used to filter out false positive results. The MS, MS^2^, and MS^3^ mass tolerances were set as 10 ppm, 20 ppm, and 0.6 Da, respectively. The XlinkX detect program (Thermo Scientific) was used to search MS^2^ data and identify the list of putative DSSO‐interlinked products based on their unique DSSO fragmentation patterns. Monoisotopic masses and charges of parent ions measured in MS^3^ scans for those putative crosslinked peptides were further validated and scored by XlinkX. The final results were confirmed by manual inspection of the MS^2^ and MS^3^ spectra, respectively. The MS data were deposited in the proteomics identifications (PRIDE) database with accession number PXD019868 and PXD019944. The lysines crosslinked with scFVs were highlighted on the structure of *h*CD38‐ECD (PDB 1yh3)^[^ ^34^ ^]^ for the epitope analyses. The protein structure was displayed by PyMOL.

##### In Vivo Daudi Cell‐Derived Xenograft Studies

Animal studies were conducted in accordance with approved IACUC protocols at WuXi Apptec, Suzhou, China. Six to eight‐week‐old female NOG (NOD.Cg‐Prkdc^scid^Il2rg^tm1Sug^/JicCrl) immunodeficient mice were used in this study. To monitor tumor growth and antitumor effect of CAR‐T cells, 1 × 10^6^ luciferase‐transduced Daudi cells were implanted in mice by intravenous (i.v.) tail injection. 7 d after implantation tumors became detectable by bioluminescence imaging (BLI). Mice were divided into two equal groups, and received mock T cells or RP02 CAR‐T cells (1 × 10^7^/mouse) via i.v. tail injection. Thereafter, the bioluminescence imaging signal persistence was measured by an IVIS spectrum imaging system (PerkinElmer) once per week until the experiment was completed.

##### Statistical Analyses

All experimental results were indicated as mean ± s.e.m. unless stated otherwise. Data analysis was performed using regular one‐way or two‐way analysis of variance (ANOVA) for comparisons of more than two groups. When only two groups were compared, statistical significance was assessed with an unpaired Student's *t*‐test. All statistics were carried out with GraphPad Prism 8. Significance was considered as *p < 0.05; **p < 0.01; ***p < 0.001; ****p < 0.0001.

## Conflict of Interest

The authors declare no conflict of interest.

## Author Contributions

P.M., P.R., C.Z. and J.T. contributed equally to this work. P.M., G.Y., and R.A.L. conceived the study. P.M., P.R., C.Z., J.T., K.F., Z.Y., G.L., X.Z., and C.M. performed the CAR selection and functional study. P.M., W.Z., and W.C. performed the crosslink analyses. R.A.L., G.Y., X.H., W.S., P.M., and P.R. wrote the paper.

## Supporting information

Supporting Information
