# Supplementary material for: Avidity‐Based Selection of Tissue‐Specific CAR‐T Cells from a Combinatorial Cellular Library of CARs
Source: Adv Sci (Weinh). 2021 Jan 29;8(6):2003091. doi: 10.1002/advs.202003091 (PMC7967050; doi:10.1002/advs.202003091)
Supplement: Supplementary file 1 — Supporting Information [file ADVS-8-2003091-s001.pdf]

© 2020 Wiley-VCH GmbH

ADVANCED SCIENCE
Open Access

Supporting Information

for *Adv. Sci.,* DOI: 10.1002/advs.202003091

Avidity-based selection of tissue-specific CAR-T cells from a combinatorial cellular library of CARs

Peixiang Ma[#], Ping Ren[#], Chuyue Zhang[#], Jiaxing Tang[#], Zheng Yu, Xuekai Zhu, Kun Fan, Guanglei Li, Wei Zhu, Wei Sang, Chenyu Min, Wenzhang Chen, Xingxu Huang, Guang Yang[*], Richard A Lerner[*]

Supporting Information

Avidity-based selection of tissue-specific CAR-T cells from a combinatorial cellular library of CARs

Peixiang Ma[#], Ping Ren[#], Chuyue Zhang[#], Jiaxing Tang[#], Zheng Yu, Xuekai Zhu, Kun Fan, Guanglei Li, Wei Zhu, Wei Sang, Chenyu Min, Wenzhang Chen, Xingxu Huang, Guang Yang[*], Richard A Lerner[*].

Supplementary Figures:

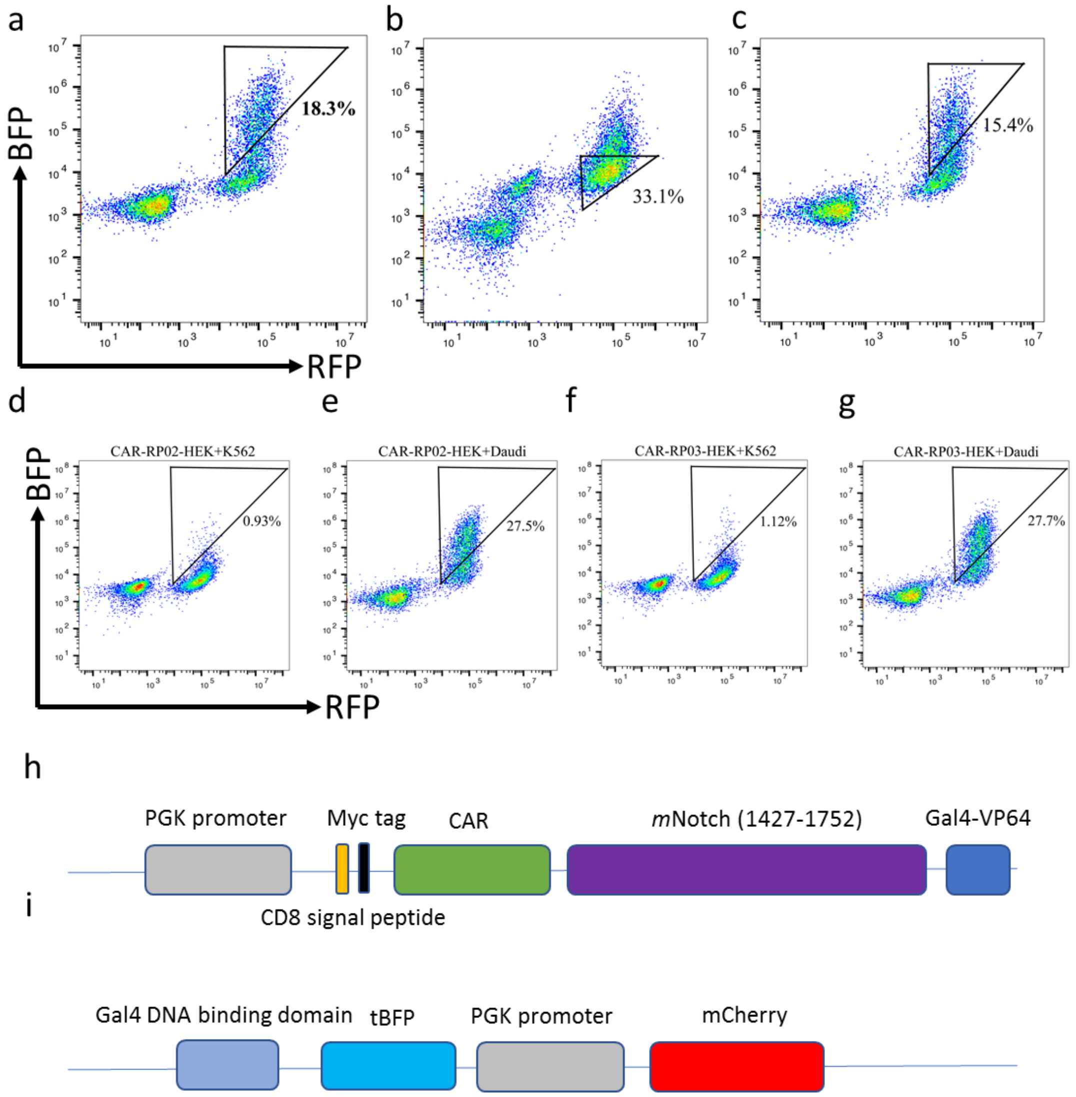

Figure S1. Proof of concept of CAR selection using cell-cell interaction. (a) The high-affinity scFV 028 was spiked into a single chain fragment variable (scFv) antibody library at a ratio of 1:10^4. **(b)** In the positive selection group, the CAR-cell library was activated by K562-CD38 cells. **(c)** In the negative-positive selection group, initially healthy PBMCs with low expression of CD38 were used to activate the CAR-cell library. The activated cells were discarded as they were the result of activation by non-tumor cells. The remaining, non-activated cells were collected and exposed to K562-CD38 cells for activation. The positive cells were collected for sequencing. RP02-CAR synNotch was expressed in HEK-293 cells, then co-cultured with $CD38^-$ (K562) cells **(d)** and $CD38^+$ (Daudi) cells **(e)**. RP03-CAR synNotch was

expressed in HEK-293 cells, then co-cultured with $CD38^-$ (K562) cells **(f)** and $CD38^+$ (Daudi) cells **(g)**. **(h)** The CAR-synNotch construct consists of an N-terminal extracellular domain containing a *h*CD8α signaling peptide, a myc peptide, and a CAR; a transmembrane domain consisting of *m*Notch1 core (from 1427 to 1752 amino acids); and an intracellular domain consisting of Gal4-VP64. (**i**) The effector construct was designed to monitor chimeric Notch signaling, in which synNotch activation resulted in Gal4-VP64 release and, subsequently, BFP expression through interaction between the released Gal4-VP64 and the Gal4 DNA binding domain. A *m*Cherry fluorescent protein was used in the construct as an internal transduction marker.

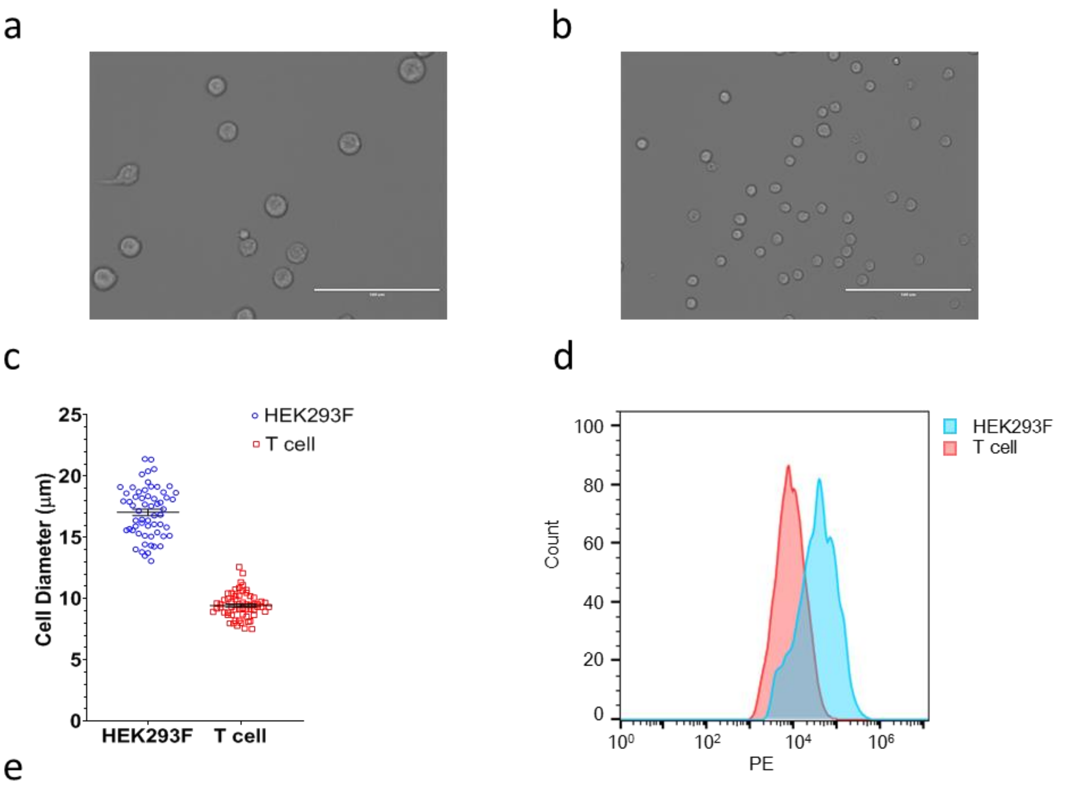

e

Cells	Diameter (μm)	Surface Area (μm²)	MFI	Density (MFI/μm²)
HEK293F	17.1 ± 0.3	918.2 ± 32.4	42832.5 ± 1348.5	46.6 ± 2.8
T Cell	9.4 ± 0.1	277.4 ± 5.9	11955.4 ± 116.4	43.1 ± 1.3

Figure S2 | Comparison of CAR density on the cell surface of HEK293F and human T cells. (a) Representative images of HEK293F cells grown in suspension. Scale bar is 100 μm. **(b)** Representative images of human T cells grown in suspension. Scale bar is 100 μm. **(c)** Diameter of HEK293F and primary T cells. The diameter of HEK293F is 17.1±0.3 μm and primary T is 9.4±0.1 μm. (n=60) **(d)** Cell surface synNotch on HEK293F and primary T cells was analyzed using PE-labelled rabbit anti- myc antibody. **(e)** Comparison of cell surface density of synNotch on HEK293F and primary T cells.

a

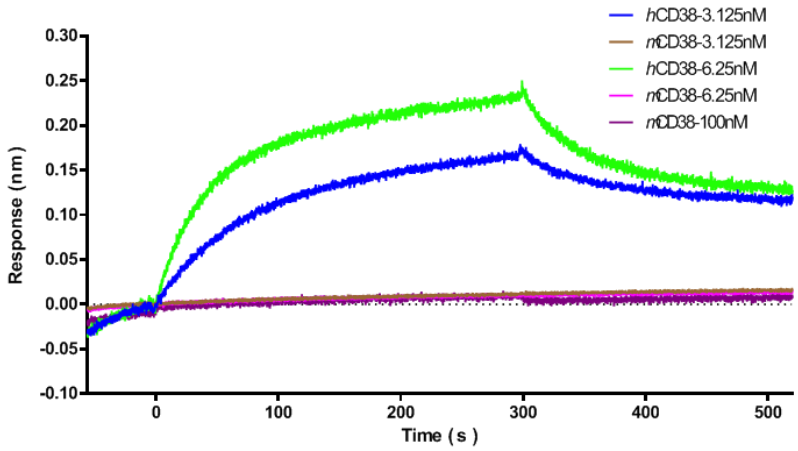

b

```
hCD38-ECD      -VPRWRQQWSGPGTTKRFPETVLARCVKYTEI-HPEMRHVDCQSVWDAFKGAFISKHPCN 58
mCD38-ECD      LRPRSLLVWTGEPTTKHFSDIFLGRCLIYTQILRPEMRDQNCQEILSTFKGAFVSKNPCN 60
                 **    *:*  ***:* : .*.**: **:* :****.  :**.: . :*****:**:***
hCD38-ECD      ITEEDYQPLMKLGTQTVPCNKILLWSRIKDLAHQFTQVQRDMFTLEDTLLGYLADDLTWC 118
mCD38-ECD      ITREDYAPLVKLVTQTIPCNKTLFWSKSKHLAHQYTWIQGKMFTLEDTLLGYIADDLRWC 120
               **.*** **:** ***:**** *:**: *.****:* :* .***********:**** **
hCD38-ECD      GEFNTSKINYQSCPDWRKDCSNNPVSVFWKTVSRRFAEAACDVVHVMLNGSRSKIFDKNS 178
mCD38-ECD      GDPSTSDMNYVSCPHWSENCPNNPITVFWKVISQKFAEDACGVVQVMLNGSLREPFYKNS 180
               *: .**.:** ***.* ::* ***::****.:*::*** **.**:******  : * ***
hCD38-ECD      TFGSVEVHNLQPEKVQTLEAWVIHGGREDSRDLCQDPTIKELESIISKRNIQFSCKNIYR 238
mCD38-ECD      TFGSVEVFSLDPNKVHKLQAWVMHDIEGASSNACSSSSLNELKMIVQKRNMIFACVDNYR 240
               *******..*:*:**:.*:***:*. .   * : *..  :::**: *:.***: *:* : **
hCD38-ECD      PDKFLQCVKNPEDSSCTSEI 258
mCD38-ECD      PARFLQCVKNPEHPSCRLNT 260
               * :*********. **  :
```

Figure S3 | Interactions between purified scFv antibodies and *h*CD38-ECD / *m*CD38-ECD proteins. **(a)** Comparison of binding affinities of RP02 and RP03 antibodies to *h*CD38-ECD / *m*CD38-ECD by surface plasmon resonance (SPR) measurement. **(b)** Sequence alignment between *h*CD38-ECD and *m*CD38-ECD.

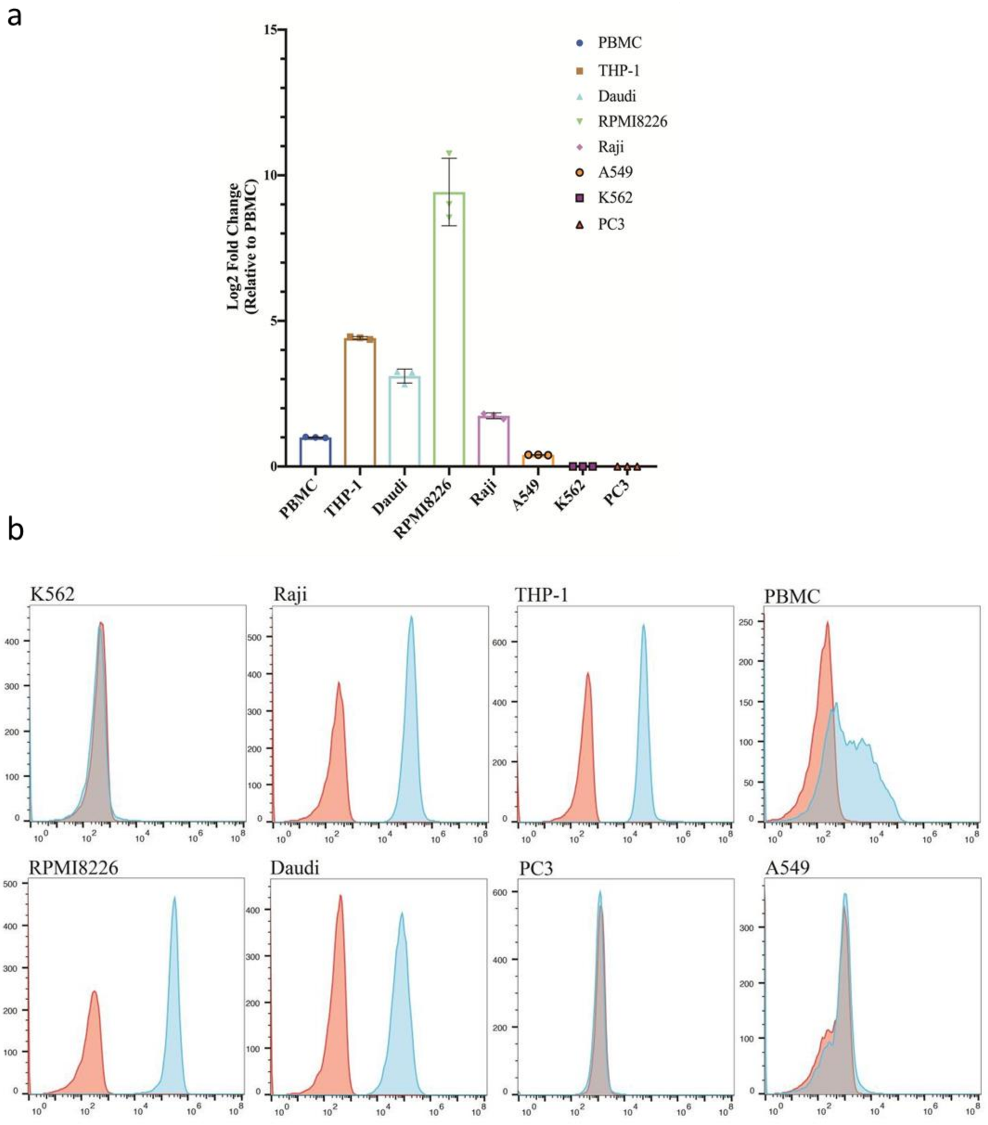

Figure S4 丨 CD38 expression in different cells. (a) Relative expression levels of CD38 in indicated cells were measured by real-time quantitative reverse transcription PCR (qRT-PCR). The results were normalized to GAPDH mRNA levels and represent the means ± SEM. (n = 3) **(b)** Cell surface expression of CD38 was evaluated by FACS.

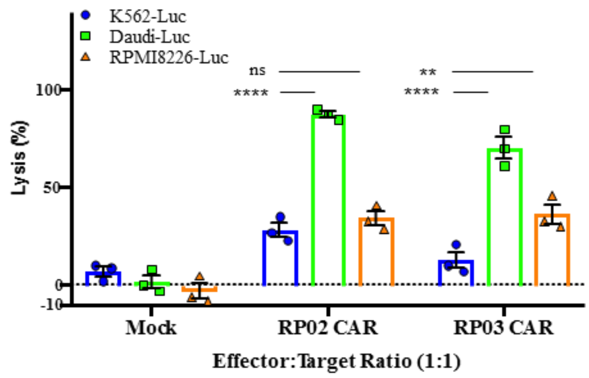

Figure S5 | Evaluation of anti-tumor efficiency of RP02 CAR-T and RP03 CAR-T.

In contrast to results seen with mock T cells, co-culture of $CD38^+$ target tumor cells (Daudi and RPMI8226) with RP02 or RP03 anti-CD38 CAR-T cells for 24 hours at an E:T ratio of 1:1 resulted in significant cytotoxicity. Data are represented as the mean ± s.e.m. of $n = 3$ technical replicates. Significance was considered as $*p < 0.05$; $**p < 0.01$; $***p < 0.001$; $****p < 0.0001$.

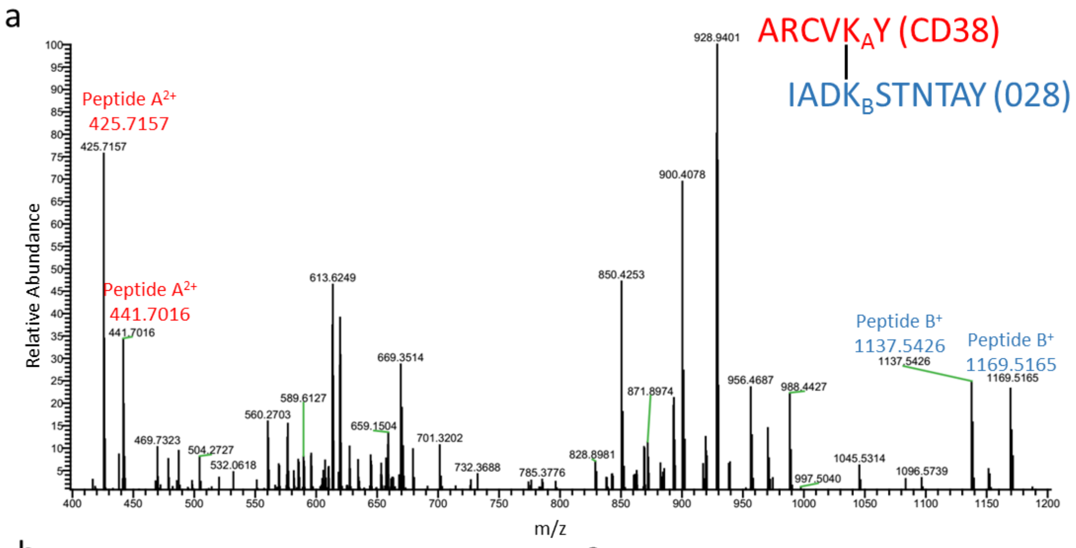

b

MS^3 of ARCVKY

#1	b^+	Seq.	y^+	#2
1	---	A	---	6
2	228.05	R	668.35	5
3	388.23	C	512.25	4
4	487.31	V	409.24	3
5	558.7	K	364.3	2
6	---	Y	182.08	1

c

MS^3 of IADKSTNTAY

#1	b^+	Seq.	y^+	#2
1	---	I	---	10
2	185.1285	A	970.44	9
3	300.1554	D	899.41	8
4	428.2504	K	766.45(-H_2O)	7
5	515.2824	S	639.26(-NH_3)	6
6	616.3301	T	551.47(-H_2O)	5
7	730.373	N	451.379(-NH_3)	4
8	831.4207	T	354.16	3
9	902.4578	A	253.11	2
10	---	Y	182.08	1

Figure S6 | 028 scFv cross-linked with *h*CD38-ECD via IADKSTNTAY and ARCVKY peptides. (**a**) Secondary MS showing cross-linked peptides ARCVKY-IADKSTNTAY. (**b**) Peptide mass fingerprint for ARCVKY. (**c**) Peptide mass fingerprint for IADKSTNTAY. The MS data were deposited in the proteomics identifications (PRIDE) database with accession number PXD019944.

a

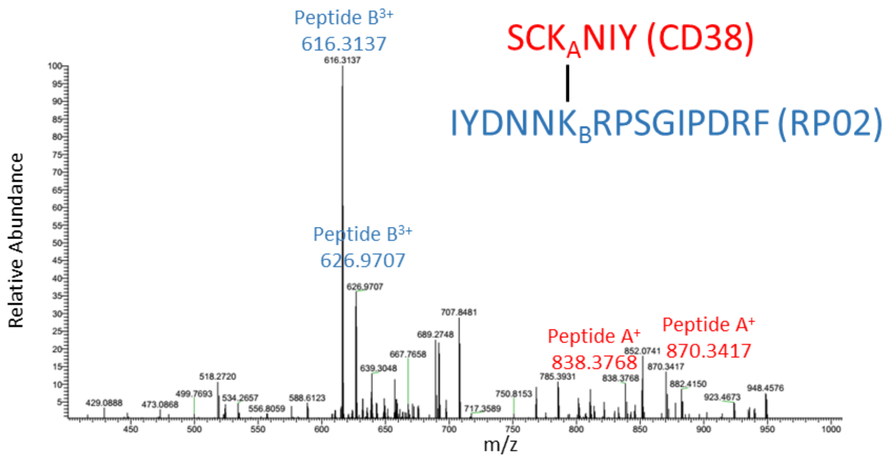

b

MS3 of SCKNIY

#1	b+	Seq.	y+	#2
1	---	S	---	6
2	191.0485	C	640.3123	5
3	301.13(-H_2O)	K	537.3031	4
4	433.1864	N	409.4	3
5	528.26(-H_2O)	I	295.1652	2
6	---	Y	182.0812	1

c

MS3 of IYDNNKRPSGIPDRF

#1	b+	Seq.	b+	b2+	#2
1	114.09	I			15
2	277.15	Y	1678.82	883.08	14
3	392.18	D	1515.76	801.6	13
4	506.22	N	1400.73	743.93	12
5	620.26	N	1286.69	670.94	11
6	785.59(-NH_3)	K	1172.65	614.2	10
7	904.46	R	1044.55	522.78	9
8	1069.57(-H_2O)	P	888.46	444.73	8
9	1088.54	S	791.4	396.2	7
10	1234.54	G	704.37	352.68	6
11	1258.65	I	647.35	324.17	5
12	1355.71	P	534.43	267.63	4
13	1470.73	D	437.21	219.11	3
14	1626.83	R	322.18	161.59	2
15		F	166.08	83.54	1

Figure S7 | RP02 scFv cross-linked with *h*CD38-ECD via IYDNNKRPSGIPDRF and SCKNIY peptides. (**a**) Secondary MS showing cross-linked peptides SCKNIY-IYDNNKRPSGIPDRF. (**b**) Peptide mass fingerprint for SCKNIY. (**c**) Peptide mass fingerprint for IYDNNKRPSGIPDRF. The MS data were deposited in the proteomics identifications (PRIDE) database with accession number PXD019868.

a

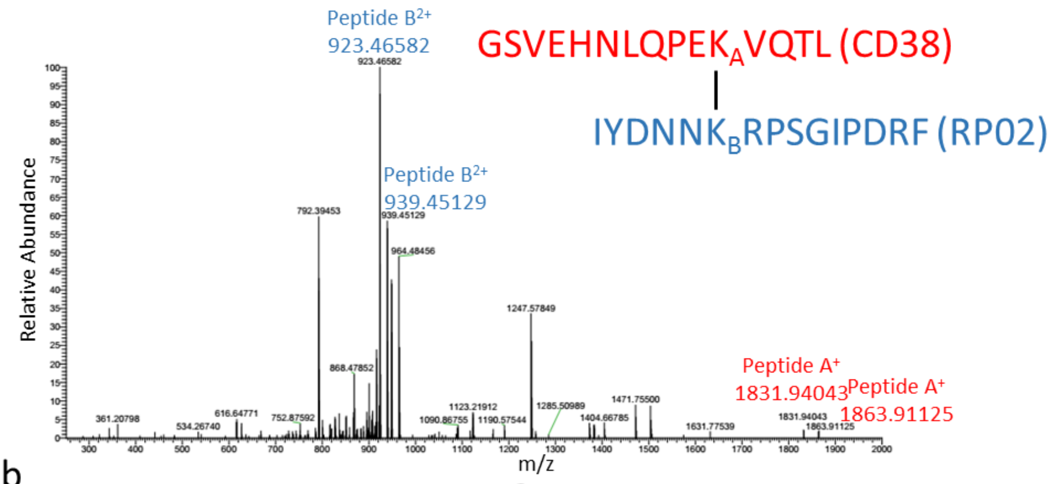

b

MS³ of GSVEVHNLQPEKVQTL

#1	b⁺	Seq.	y⁺	y²⁺	y³⁺	#2
1	58.02	G				16
2	145.06	S	1720.92	860.96	574.31	15
3	244.12	V	1633.89	817.44	545.3	14
4	373.17	E	1534.82	767.91	512.27	13
5	472.24	V	1405.77	703.39	469.26	12
6	591.28 (-H_2O)	H	1306.71	653.85	436.24	11
7	723.34	N	1169.65	585.32	390.55	10
8	836.42	L	1055.6	528.3	352.54	9
9	964.56	Q	942.52	471.76	314.84	8
10	1061.53	P	814.46	407.73	272.16	7
11	1190.52	E	717.41	359.21	239.8	6
12	1405.06	K	588.37	329.19(-NH_3)	196.79	5
13	1503.76	V	460.27	230.64	154.09	4
14	1545.8	Q	343.19(-H_2O)	181.1	121.07	3
15	1646.84	T	233.14	117.07	78.38	2
16		L	132.1	66.55	44.7	1

c

MS³ of IYDNNKRPSGIPDRF

#1	b⁺	Seq.	b⁺	b²⁺	#2
1	114.09	I			15
2	277.07	Y	1678.82	883.24	14
3	392.18	D	1515.76	801.6	13
4	506.22	N	1400.73	728.03	12
5	620.26	N	1286.69	671.33	11
6	785.5(-NH_3)	K	1172.65	613.9	10
7	990.56	R	1044.85	523.05	9
8	1069.57(-H_2O)	P	888.72	444.73	8
9	1088.54	S	791.4	396.2	7
10	1199.83	G	704.37	352.68	6
11	1258.65	I	647.35	324.17	5
12	1355.71	P	534.26	267.63	4
13	1470.73	D	437.21	219.11	3
14	1626.83	R	322.18	161.59	2
15		F	166.08	83.54	1

Figure S8 | RP02 scFv cross-linked with *h*CD38-ECD via IYDNNKRPSGIPDRF and GSVEVHNLQPEKVQTL peptides. (**a**) Secondary MS showing cross-linked peptides GSVEVHNLQPEKVQTL-IYDNNKRPSGIPDRF. (**b**) Peptide mass fingerprint for GSVEVHNLQPEKVQTL. (**c**) Peptide mass fingerprint for IYDNNKRPSGIPDRF. The MS data were deposited in the proteomics identifications (PRIDE) database with accession number PXD019868.

a

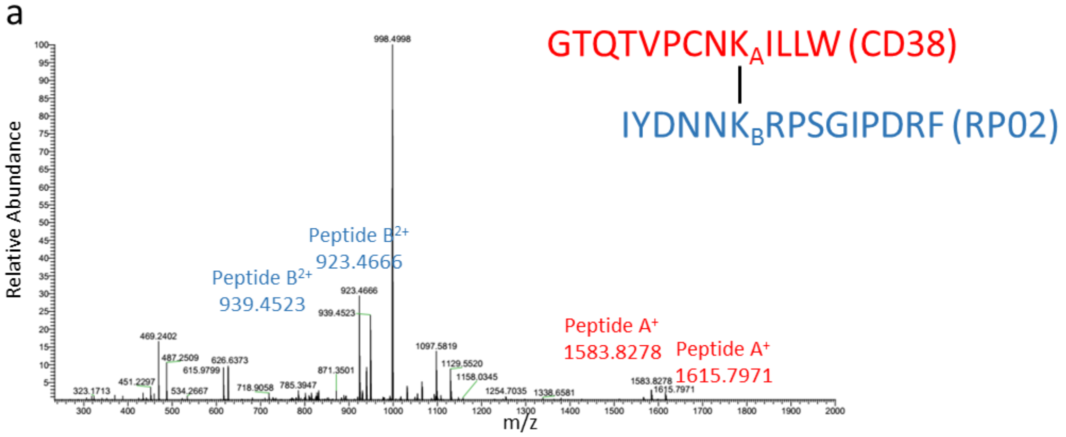

b

MS³ of GTQTVPCNKILLW

#1	b⁺	Seq.	y⁺	#2
1	---	G	---	13
2	159.15	T	1415.7715	12
3	287.135	Q	1314.7239	11
4	388.1827	T	1186.6653	10
5	487.2511	V	1085.6176	9
6	584.3039	P	986.5492	8
7	687.313	C	889.28	7
8	801.356	N	840.38	6
9	929.4509	K	726.5	5
10	1042.535	I	544.3493	4
11	1155.6191	L	431.2653	3
12	1268.7031	L	318.1812	2
13	---	W	205.0972	1

c

MS³ of IYDNNKRPSGIPDRF

#1	b⁺	Seq.	b⁺	b²⁺	#2
1	114.09	I			15
2	277.07	Y	1678.82	883.24	14
3	392.18	D	1556.8	801.6	13
4	506.22	N	1400.73	744.18	12
5	620.26	N	1286.69	671.33	11
6	785.5	K	1172.65	613.9	10
7	990.56	R	1044.85	523.05	9
8	1069.57	P	888.5	444.73	8
9	1088.54	S	791.4	396.2	7
10	1199.83	G	704.37	352.68	6
11	1258.65	I	647.35	324.17	5
12	1355.71	P	534.32	267.63	4
13	1524.79	D	437.21	219.11	3
14	1626.83	R	322.12	161.59	2
15		F	166.08	83.54	1

Figure S9 | RP02 scFv cross-linked with *h*CD38-ECD via IYDNNKRPSGIPDRF and GTQTVPCNKILLW peptides. (**a**) Secondary MS showing cross-linked peptides GTQTVPCNKILLW-IYDNNKRPSGIPDRF. (**b**) Peptide mass fingerprint for GTQTVPCNKILLW. (**c**) Peptide mass fingerprint for IYDNNKRPSGIPDRF. The MS data were deposited in the proteomics identifications (PRIDE) database with accession number PXD019868.

	CDR3
028-V_H	EPGERDPD-AVDI
RP02-V_H	ED----YYYYMDV
RP03-V_H	DGLPRWNDIWFDL

	CDR3
028-V_L	QQYNS--YPLT
RP02-V_L	GTWDSSLSAGV
RP03-V_L	TSFTT-FKTWV

Figure S10 | Sequence alignment of variable domains. The CDR3 sequences of 028, RP02 and RP03 scFvs were aligned. The sequences were numbered using the Kabat numbering scheme.
